# Optimization of a VOC Sensor with a Bilayered Diaphragm Using FBAR as Strain Sensing Elements

**DOI:** 10.3390/s17081764

**Published:** 2017-08-01

**Authors:** Huihui Guo, Aohui Guo, Yang Gao, Tingting Liu

**Affiliations:** 1 School of Information Engineering, Southwest University of Science and Technology, Mianyang 621010, China; ljttlf@163.com; 2 Xizang Agriculture and Animal Husbandry College, Linzhi 860000, China; manyoudemeng@163.com; 3 Institute of Electronic Engineering, China Academy of Engineering Physics, Mianyang 621919, China; yanggao@caep.cn

**Keywords:** VOC sensor, FBAR, FEA, bilayered diaphragm

## Abstract

Film bulk acoustic resonators (FBARs) are widely applied in mass bio-sensing and pressure sensors, owing to their extreme sensitivity and integration ability, and ability to miniaturize circuits. A volatile organic compound (VOC) sensor with a polymer-coated diaphragm, using FBARs as a strain sensing element is proposed and optimized. This vapor sensor is based on organic vapor-induced changes of mechanical deformation of the micro-diaphragm. The four FBARs are located at the edge of the bi-layer diaphragm comprising silicon nitride and silicon oxide for strain extraction. In this work, the strain distribution of the FBAR area under vapor loads is obtained using the finite element analysis (FEA) and the response frequency changes of the FBARs under vapor loads are obtained based on both the first-principle methods to deduce the elastic coefficient variation of aluminum nitride film in FBARs under the bending stresses and the Mason equivalent circuit model of the sensor using ADS software. Finally, optimizations are performed on both the bilayered diaphragm structure and sensing film. The diaphragm with a 0.7 μm silicon nitride layer and a 0.5 μm silicon oxide layer are considered to be the optimized design. The optimal coverage area of the sensing film for the diaphragm is around 0.8.

## 1. Introduction

Film bulk acoustic resonators (FBARs) have experienced rapid development in the past 15 years, owing to the skyrocketing development of mobile communication [1]. FBARs are widely applied in small phone filters and duplexers. Recently, FBARs have also demonstrated promise in sensor applications such as bio-sensing [2], mass [3] and pressure sensors [4] owing to their miniature size which favors easy integration potential with complementary metal-oxide-semiconductor (COMS) circuits, extreme sensitivity, and easy arraying for multi-channel functioning. The basic configuration of FBARs is a membrane structure consisting of a piezoelectric thin film sandwiched between two electrodes. A membrane can be formed either by etching the Si substrate from the back or from the front surface by etching a pre-buried sacrificial layer. The released membrane structure makes FBARs more sensitive to external forces, which then have great potential to realize high sensitivity [1]. The sensitivity of the FBAR force sensor relies on stress-induced changes of thickness and longitudinal acoustic velocities of the piezoelectric thin film in FBARs. The high operation frequency of FBARs is beneficial in obtaining high sensitivity and high resolution for strain extraction. Therefore, the FBAR has great potential as a sensing element in high precision mechanics sensors.

As is known, volatile organic compounds (VOCs) are well recognized as serious environment pollutants and are considered as a source of danger to human health [5,6]. Therefore, small size, high sensitivity and easy integration with the CMOS circuit of vapor sensors is urgently required to detect VOC vapor. Sensors based on vapor-induced volume expansion of the sensing film coated on the micro-diaphragm embedded with a piezoresistor have been developed in our previous work [7,8]. Experiments show that those sensors have some good characteristics, for example, good linearity, small size, low power consumption and CMOS compatibility. Furthermore, the best sensitivity of those sensors for chloroform is extracted as 1.41 μV/V/ppm and the minimum detectable concentration of chloroform vapor is only 10 ppm [8]. According to the Chinese national indoor air quality standard (GB/T 18883) for VOC vapor, the concentration of formaldehyde, benzene and xylene must be less than 0.1 mg/m^3^ (14 ppm), 0.11 mg/m^3^ (30 ppm) and 0.2 mg/m^3^ (24 ppm), respectively. Thus, the sensitivity and minimum detectable concentration of those vapor sensors could not meet the monitoring requirement of the indoor pollutants. Therefore, we have to develop greater sensitivity and a lower detection limit of the sensor for monitoring indoor pollutants.

**Figure 1:**
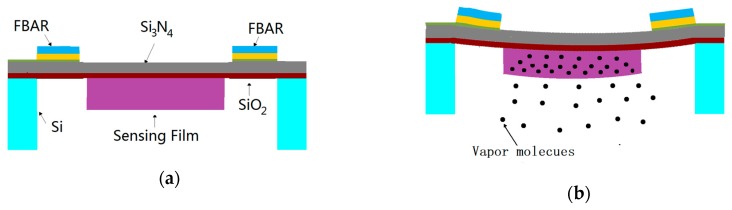
Schematic of the volatile organic compound (VOC) sensor: (**a**) Cross section view; (**b**) Drawing of the working principle.

Due to the high performance of the FBAR as a force sensor, vapor sensors using the FBAR instead of the piezoresistor as a strain sensing element have great potential to improve the sensitivity and reduce the detection limit for detecting indoor pollutants. A VOC sensor based on a polymer-coated diaphragm embedded with FBAR is presented. The working principle of this sensor is explained as follows: the swelling of the polymer film due to the absorption of vapor molecules from the atmosphere causes the deformation of the micro-diaphragm and the bending stress loads on the piezoelectric film of the FBAR located at the edge of the micro-diaphragm. Then, the longitudinal acoustic wave velocity in the piezoelectric layer is changed under bending stress; subsequently, this causes the resonant frequency shift of the FBAR. The schematic drawing of the working principle is shown in Figure 1. Finally, the resonant frequency shift can be read out or measured by a vector network analyzer. The VOC sensor is composed of a circular diaphragm structure embedded with the FBAR and a sensing film coated on the Micro-diaphragm. To improve the performance of this sensor, the design parameters of the diaphragm structure and sensing film should be optimized separately.

## 2. Diaphragm Optimization

In this paper, the FBAR is a membrane structure consisting of a piezoelectric thin film sandwiched between two electrodes, and the member is formed by etching the Si substrate from the back as shown in Figure 1a. The silicon nitride (Si_3_N_4_) thin film is a good support layer for the FBAR owing to its good physical properties which have no side effect on the FBAR resonant frequency [9]. However, there are some drawbacks to the Si_3_N_4_ film, such as high-quality Si_3_N_4_ film cannot grow too thick on the Si substrate due to the process constraints and the Si_3_N_4_ film is easily over-etched in the backside etching step. To solve these problems, the bilayer membrane structure comprising Si_3_N_4_ and SiO_2_ as the supporting layer for the FBAR is presented owing to the silicon oxide (SiO_2_) film which has the self-stopped characteristic in the Si DRIE process and it can also improve the temperature stability of the FBAR [9].

As is well known, the thickness of the support layer will limit the measurement range and sensitivity to stress. Due to our fabrication process constraints and the device performance considerations, high-quality SiO_2_ film cannot grow too thick on the Si substrate. In this paper, a 0.5 μm SiO_2_ layer is deposited on the Si substrate and the Si_3_N_4_ layer is further deposited as a support layer for the FBAR. Due to internal stress difference, the stress on the SiO_2_ film is compressive stress and the stress on the Si_3_N_4_ film is tensile stress. The pure SiO_2_ diaphragm suffers from buckling and wrinkling issues due to its internal compressive stress. Louliang et al. [10] discussed the buckling state of the bilayer diaphragm comprising Si_3_N_4_ and SiO_2_; they confirmed that the central deflection of the membrane with 0.5 μm SiO_2_ layer change is relatively small within the range, where the thickness of the Si_3_N_4_ layer varies from 2.5 μm to 0.7 μm. They also confirmed that the deflection jumps from around 0.4 μm to nearly 5 μm as the thickness of the Si_3_N_4_ is further thinned down from 0.7 μm to 0 μm. Thus, it can be seen that the thickness of the Si_3_N_4_ layer, as part of the support layer for the FBAR, is not less than 0.7 μm in order to retain minimized diaphragm deflection. Moreover, the sensitivity of the diaphragm to stress or pressure will be decreased with increasing the thickness of the diaphragm. To fabricate the high-performance diaphragm structure for the VOC sensor, the sensitivity and the diaphragm deflection should be considered together.

To investigate the resonant frequency of the FBAR with different thicknesses of the Si_3_N_4_ layer, the 5-layer Mason equivalent circuit model was built with the help of ADS software. The material and structure parameters of the 5-layer film were determined by both design considerations and process constraints. In this work, the four FBARs with a resonant area of 50 × 25 μm^2^ were placed at the edge of the bilayer diaphragm for strain extraction, as shown in Figure 1a. All material and structure parameters are listed in Table 1.

**Figure 2:**
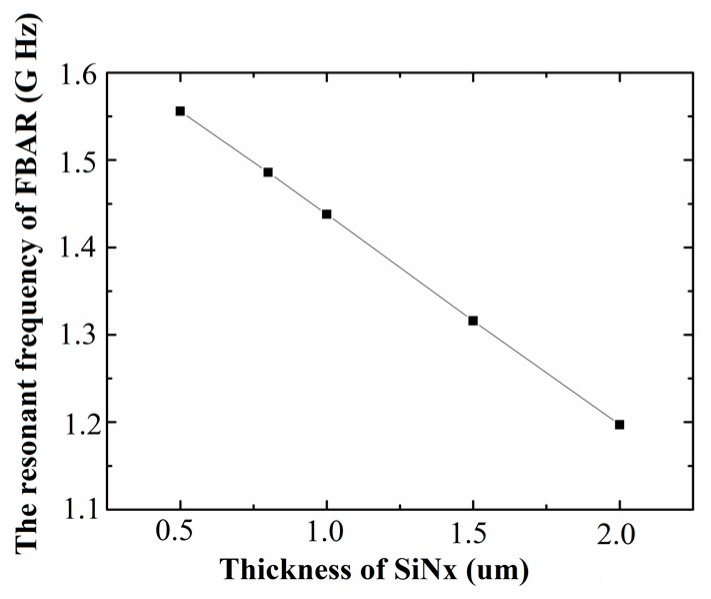
The resonant frequency of the film bulk acoustic resonator (FBAR) with different silicon nitride layers.

The resonant frequency of FBAR with different thickness of silicon nitride layer is shown in Figure 2. It can be seen that the parallel resonant frequency of FBAR from 1.575 GHz to 1.197 GHz as the thickness of the Si_3_N_4_ is increased from 0.5 μm to 2 μm. Due to the high operation frequency is beneficial in obtaining high sensitivity [1], the smaller thickness of the Si_3_N_4_ is better.

**Figure 3:**
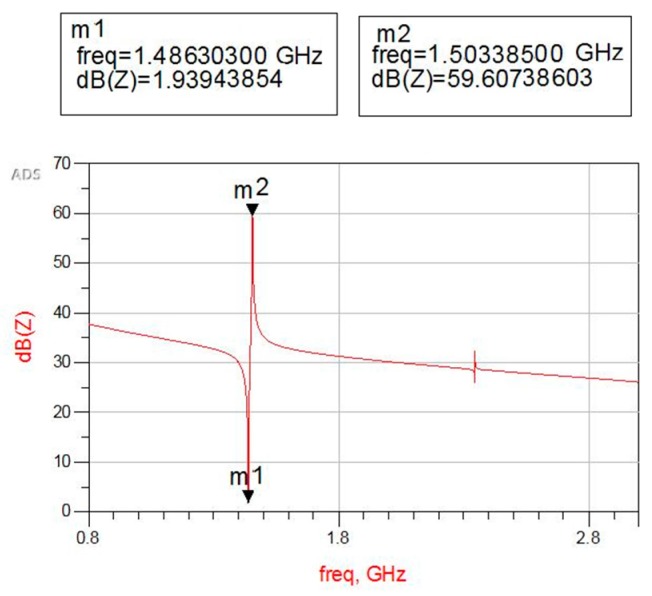
The impedance characteristic curve of the 5-layer FBAR with an optimal support layer.

Combined with the central deflection of the diaphragm caused by internal stress of the support layer, the optimal thicknesses of the Si_3_N_4_ and SiO_2_ films are 0.7 μm and 0.5 μm respectively, in order to maximize the sensitivity and also retain minimized diaphragm deflection. The simulated impedance characteristic curve of the FBAR with optimal thickness of the support layer is shown in Figure 3. Series resonant frequency of the FBAR marked m1 is about 1.486 GHz and parallel resonant frequency marked m2 is about 1.503 GHz.

## 3. Sensing Film Optimization

In this work, the vapor sensor is based on organic vapor-induced changes of mechanical deformation of the micro-diaphragm. The four FBARs are located at the edge of the diaphragm as a strain detecting element to transform the deformation into a resonant frequency shift of the FBAR. When the parameters of the diaphragm embedded with the FBAR are fixed, the parameters of the sensing layer have a great influence on the output of this VOC sensor. To investigate the relationship between the parameters of the sensing layer and the output of the vapor sensor, a model of the senor is built. In this model, the strain distribution of the FBAR area under vapor loads is obtained using finite element analysis (FEA) relying on the equivalence principle of polymer swelling which has been verified as effective in our previous work [11]. Then, the response frequency changes of the FBAR under vapor loads are obtained based on both the first-principle methods to deduce the elastic coefficient variation of the aluminum nitride film in the FBAR under the bending stresses and the Mason equivalent circuit model of the sensor using ADS software.

To obtain the strain distribution of the FBAR area under vapor loads, all material and structure parameters of the vapor sensor are listed in Table 2. In this simulation, the sensing film of VOC is PDMS film and the vapor load is chloroform. To reduce the side effect of the sensing film on the FBAR resonant frequency, the sensing film should not cover the back-side of the FBAR structure. The thicknesses of the sensing layer, Si_3_N_4_ layer and SiO_2_ layer are 20 μm, 0.7 μm and 0.5 μm, respectively. The FBAR has a resonant area of 50 × 25 μm^2^. The swelling coefficient of PDMS in chloroform vapor is calculated from experimental data as approximately 0.9 × 10^−5^/ppm and the coefficient of linear thermal expansion of PDMS is approximately 3 × 10^−4^/°C in Reference [12]. An equivalent relation between temperature load and vapor concentration load is ΔT=0.03ΔC^[11]^. The loads are added on the sensing film using reduced gradient. Here, the coverage area χ of the PDMS film is defined as the ratio between the PMDS film area to the diaphragm area in order to investigate the influence of the sensing film area on the output of the sensor.

**Figure 4:**
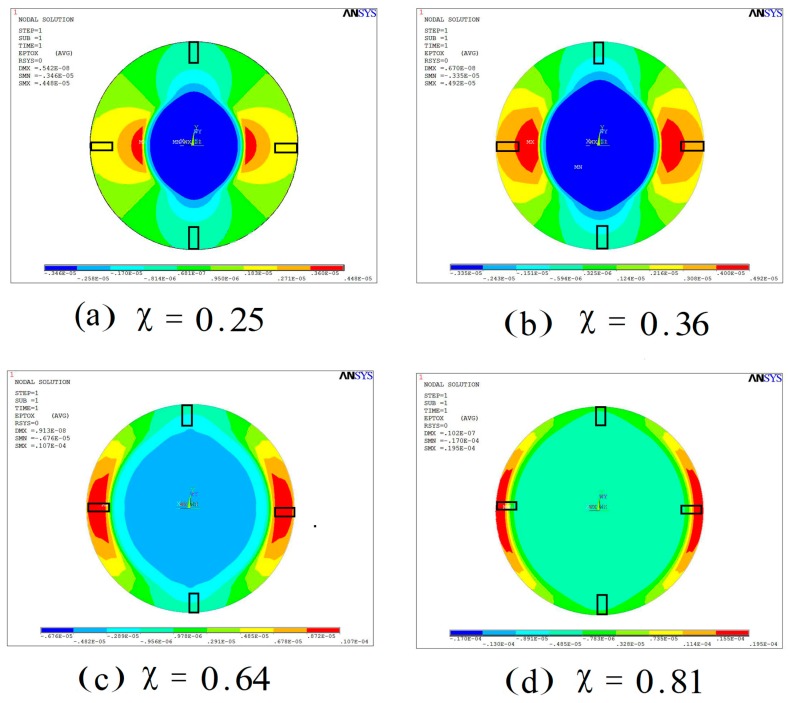
The strain distribution of the FBAR area with different coverage areas of sensing film under the same load: (**a**) χ = 0.25; (**b**) χ = 0.36; (**c**) χ = 0.64 (**d**) χ = 0.81.

Then, the bending strain distribution of the FBAR area with a different coverage area χ of the PDMS film under the same load is shown in Figure 4. It can be seen that the maximum strain area is moving toward the edge of the diaphragm with increasing coverage area of the sensing film. However, the maximum strain area is becoming smaller and smaller when the coverage area χ is greater than 0.64. Because the maximum strain area decreases quickly from the edge to the center of the diaphragm as shown in Figure 4d, the average strain of the FBAR area will decrease because part of the FBAR is not in maximum strain area. Therefore, the optimal value of the coverage area χ should exist.

**Figure 5:**
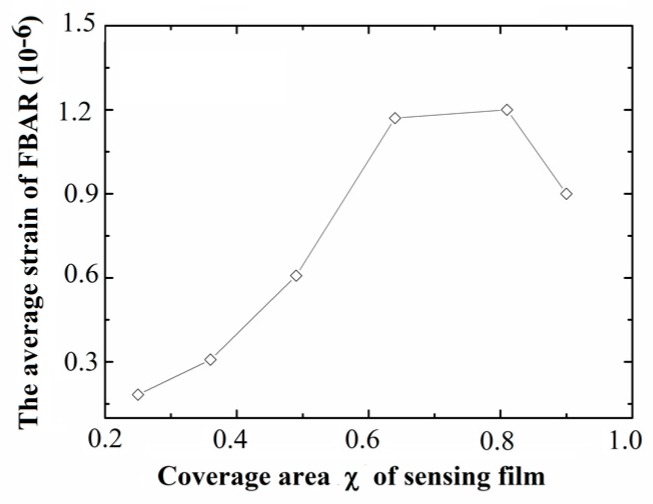
The average strain of the FBAR area with different coverage areas of sensing film under the same load.

The average strain of the FBAR area with different coverages of sensing film under the same load are obtained and calculated using the same simulation method. The average strain curve of the FBAR under the same load is shown in Figure 5. The results show that the optimal coverage area χ of the sensing film for this vapor sensor is around 0.8.

## 4. Response Characteristic of Sensor

With the help of first-principle calculation methods, the elastic constant-stress load characteristics of the wurtzite AlN film can be obtained with good accuracy. Zhifan Wang et al. [13] performed extensive first-principle studies to discuss the effect of uniaxial mechanical pressure on the structural and physical properties of AlN; then, the longitudinal elastic constant $C_{33}$ and acoustic wave velocity $v_{Z}$ of AlN under uniaxial pressure are given respectively as:

(1) $$C_{33}=357.4+3.15p_{/}-0.12p_{/}^{2}$$

(2) $$v_{z}=10984.57+25.25p_{/}-1.17p_{l}^{2}$$

where $p_{/}$ is the uniaxial pressure (G Pa) in the basal plane on Wurtzite AlN film.

**Figure 6:**
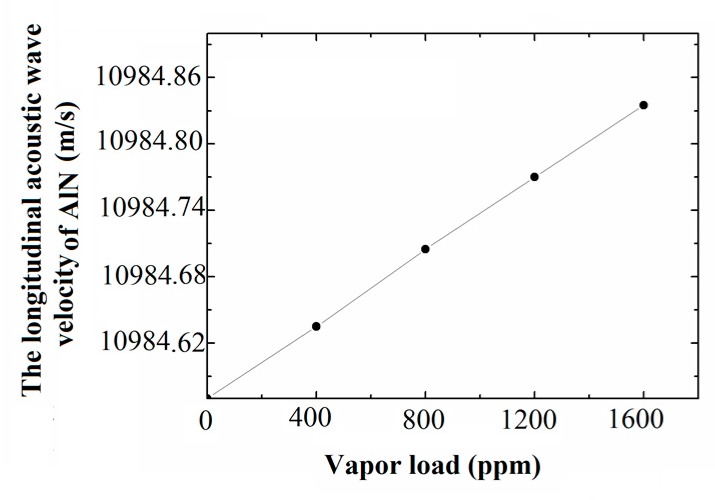
The change of longitudinal acoustic wave velocity in AlN under different vapor loads.

For qualitative analysis on the frequency response of the sensor, the radial stress of the FBAR area, as uniaxial pressure load, is applied to the Wurtzite AlN film under vapor load. To obtain the radial stress of the FBAR area under different vapor loads, the sensor with the optimal design parameters comprises a diaphragm structure and sensing film. The material and structure parameters of the sensor are shown in Table 2. Then, the value of the longitudinal acoustic wave velocity $v_{z}$ in AlN could be calculated by Equation (2). Finally, the longitudinal acoustic wave velocity curve of the AlN under different vapor loads is shown in Figure 6. The results show that the change of longitudinal acoustic wave velocity in AlN is in a good linear relationship with the concentration of chloroform vapor loads.

**Figure 7:**
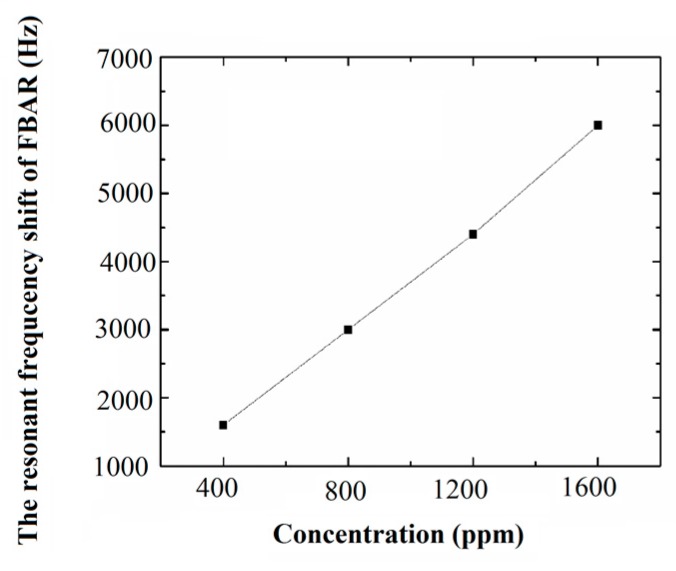
The resonant frequency change curve of the sensor for chloroform vapor.

With the help of ADS software, the five-layer Mason equivalent circuit model of the FBAR is established to obtain the resonant frequency change of the FBAR with the varied longitudinal acoustic wave velocity $v_{Z}$ in the AlN film. The resonant frequency change curve of the sensor for chloroform under different vapor concentrations is obtained, as shown in Figure 7.

The simulation results show that the VOC sensor based on the polymer-coated diaphragm using the FBAR as a strain element has relatively good linearity and good sensitivity. The sensitivity of this sensor for chloroform vapor is approximately 4 Hz/ppm. The results also show that this type of VOC sensor can basically meet the monitoring requirements for the indoor pollutants.

## 5. Conclusions

This paper optimized a VOC sensor based on a polymer-coated diaphragm using a FBAR as the strain element. In order to maximize the strain sensitivity of the FBAR, the thickness of the bilayer diaphragm comprising Si_3_N_4_ and SiO_2_ has been optimized with the help of Finite element analysis and the Mason equivalent circuit model for the 5-layer FBAR. As a result, the diaphragm with a 0.7 μm Si_3_N_4_ layer and a 0.5 μm SiO_2_ layer are considered to be the optimized design for strain extraction. In addition, the sensing film coverage area is also optimized as 0.8. Finally, the frequency response characteristic of this sensor is obtained. The results show that this sensor has relatively good linearity and good sensitivity. The sensitivity of this sensor for chloroform vapor is approximately 4 Hz/ppm.

## Figures and Tables

**Table 1:** Material and structure parameters of the FBAR.

Material	Density (g/cm^3^)	Dielectric Loss (dB/m)	Acoustic Impedance (kg/m^2^ s)	Longitudinal Acoustic Wave Velocityy (m/s)	Film Thickness (μm)
SiO_2_	2.3	-	1.25 × 10^7^	6253	0.5
Si_3_N_4_	3.25	-	3.6 × 10^7^	11,000	0.7
Pt	21.45	-	6.0 × 10^7^	2789	0.1
AlN	3.2	800	3.7 × 10^7^	10,984.57	1
Al	2.7	7500	1.76 × 10^7^	6526	0.9

**Table 2:** Material and structure parameters of the sensor modeling.

Material	Elastic Modulus (G Pa)	Poisson Ratio (μ)	Density (g/cm^3^)	Coefficient of Thermal Expansion (10^−6^/°C)	Film Thickness (μm)	Film Radius (μm)
SiO_2_	2.3	0.17	2.3	0.5	0.5	300
Si_3_N_4_	3.25	0.28	3.25	2.35	0.7	300
PDMS	0.007	0.48	0.96	300	20	240

PDMS—Polydimethylsiloxane.
